# PLSDB: advancing a comprehensive database of bacterial plasmids

**DOI:** 10.1093/nar/gkab1111

**Published:** 2021-11-25

**Authors:** Georges P Schmartz, Anna Hartung, Pascal Hirsch, Fabian Kern, Tobias Fehlmann, Rolf Müller, Andreas Keller

**Affiliations:** Chair for Clinical Bioinformatics, Saarland University, 66123 Saarbrücken, Germany; Chair for Clinical Bioinformatics, Saarland University, 66123 Saarbrücken, Germany; Chair for Clinical Bioinformatics, Saarland University, 66123 Saarbrücken, Germany; Department of Microbial Natural Products, Helmholtz-Institute for Pharmaceutical Research Saarland (HIPS), Helmholtz Centre for Infection Research (HZI), Campus E8 1, 66123 Saarbrücken, Germany; Chair for Clinical Bioinformatics, Saarland University, 66123 Saarbrücken, Germany; Chair for Clinical Bioinformatics, Saarland University, 66123 Saarbrücken, Germany; Department of Microbial Natural Products, Helmholtz-Institute for Pharmaceutical Research Saarland (HIPS), Helmholtz Centre for Infection Research (HZI), Campus E8 1, 66123 Saarbrücken, Germany; Department of Pharmacy, Saarland University, 66123 Saarbrücken, Germany; Chair for Clinical Bioinformatics, Saarland University, 66123 Saarbrücken, Germany; Department of Microbial Natural Products, Helmholtz-Institute for Pharmaceutical Research Saarland (HIPS), Helmholtz Centre for Infection Research (HZI), Campus E8 1, 66123 Saarbrücken, Germany

## Abstract

Plasmids are known to contain genes encoding for virulence factors and antibiotic resistance mechanisms. Their relevance in metagenomic data processing is steadily growing. However, with the increasing popularity and scale of metagenomics experiments, the number of reported plasmids is rapidly growing as well, amassing a considerable number of false positives due to undetected misassembles. Here, our previously published database PLSDB provides a reliable resource for researchers to quickly compare their sequences against selected and annotated previous findings. Within two years, the size of this resource has more than doubled from the initial 13,789 to now 34,513 entries over the course of eight regular data updates. For this update, we aggregated community feedback for major changes to the database featuring new analysis functionality as well as performance, quality, and accessibility improvements. New filtering steps, annotations, and preprocessing of existing records improve the quality of the provided data. Additionally, new features implemented in the web-server ease user interaction and allow for a deeper understanding of custom uploaded sequences, by visualizing similarity information. Lastly, an application programming interface was implemented along with a python library, to allow remote database queries in automated workflows. The latest release of PLSDB is freely accessible under https://www.ccb.uni-saarland.de/plsdb.

## Graphical Abstract

**Graphical Abstract:**
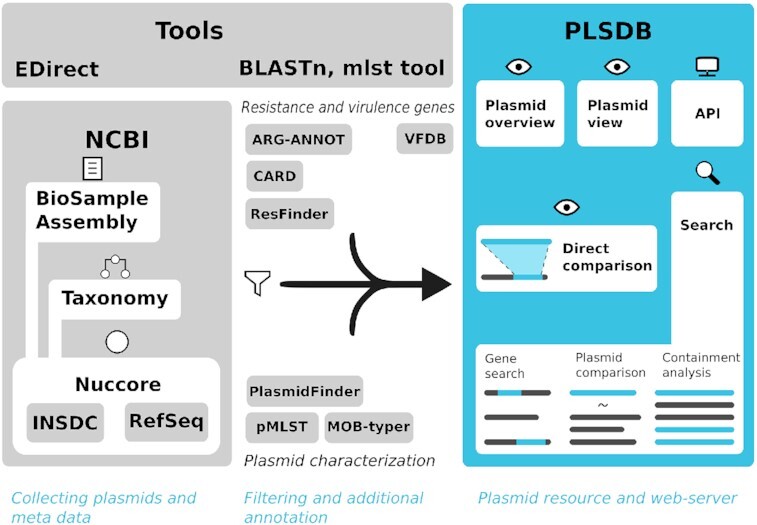
PLSDB aggregates plasmid and meta data from different public resources. This data passes through several enhanced annotation and filtering steps. Finally, the information is presented in a web-server, providing two new functionalities.

## INTRODUCTION

Plasmids are extrachromosomal DNA sequences that are short in comparison to chromosomes and frequently found in circular form within prokaryotes. They can harbor a wide range of genes such as antibiotic resistance and virulence factors (1,2). Due to the appearance of such clinically relevant phenotypes, the analysis of plasmid sequences is widely acknowledged and often performed in the context of microbiome sequencing studies (3,4). On the one hand, associative connections between clinical conditions and plasmids may allow untangling specific disease and treatment patterns. On the other hand, plasmid research furthermore plays a significant role on a population level (5). Due to several mechanisms, e.g., horizontal gene transfer via conjugation, antibiotic resistance may spread calling for a readjustment of focus in pharmaceutical research on new innovative antibiotics (6). However, to allow monitoring global distributions of plasmids within populations, a general-purpose database is required, providing easy access to previously reported plasmids. Here, PLSDB (7) supports researchers with an easy-to-use web interface since 2018.

The original PLSDB was created to complement NCBI’s plasmid collection on RefSeq, which is partially incomplete, inconsistent, lacking in functionality, and contains several chromosomal sequences. PLSDB gathers data from NCBI & INSDC based on the query formulated by Orlek A et al. (8) and adds further filtering and annotation steps. The filtering hereby focuses on deduplication, Mash distances (9), and identification of putative chromosomal sequences using 53rps genes from PubMLST (10). The additional annotations consist of resistance and virulence factors from ARG-ANNOT (11), CARD (12), ResFinder (13) and VFDB (14). Apart from the dataset, PLSDB also provides a web interface to present the data in a simple but powerful manner. A core function of PLSDB is to allow users to upload their own sequences and compare them to the database contents, thereby selecting from established search methods such as Mash (9) or blastn (15).

With the rising popularity of whole metagenome shotgun sequencing slowly superseding 16S rRNA sequencing, more plasmids are getting discovered. Furthermore, dedicated algorithms for plasmid extraction from short read sequencing are gaining attention allowing for more efficient automated analysis of sequencing data (16–18). Similarly, new databases emerge trying to manage and overcome the resulting flood of plasmid data. A new plasmid collection by Brooks et al., for example, tries to bundle NCBI plasmid information in a collection (19). Another recent database, mMGE (20) has the advantage of unifying phage and plasmid information in a single catalog. However, the database creation workflow is solely focused on the human microbiome. The COMPASS database (21), is of comparable scope to PLSDB and focuses extensively on replicon typing. Due to the scope, size, functionality, and quality of its content, PLSDB is widely used in the scientific community as a central resource for reference data on natural occurring plasmids. By focusing on this domain, the resource finds extensive usage in environmental studies (22). Further, antibiotic resistance analyses with a diverse scope profit frequently from the resistance annotations found in PLSDB (23,24). Based on aggregated feedback of this primary expert userbase, we conducted a major update. A sizable portion of the update focuses on improving future maintenance, aggregation, and quality of the data. Further, we saw this as an opportunity to implement various new utilities into the online resource (Graphical Abstract).

**Figure 1.**
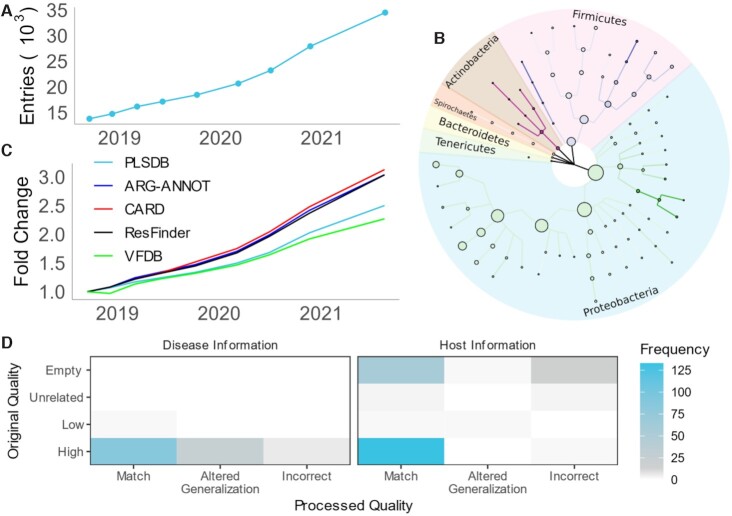
New Data of PLSDB: (**A**) Growth of the PLSDB data collection over time. (**B**) Taxonomic tree capturing the main composition of PLSDB in terms of quantity across several taxonomic ranks. Node size indicates frequency in the current database. Color fade represents relative growth compared to the first release of PLSDB. (**C**) Yearly growth of annotation data per source collection. Fold change is always computed with respect to the first release. (**D**) Manual validation of automatic preprocessing results. For each information type, annotations are compared before and after preprocessing. To generate the heatmap, all unique lowercase representatives of descriptions were extracted from the current version of PLSDB. Entries were then manually evaluated. For the preprocessed description, the comparison was drawn to the respective ontologies.

## MATERIALS AND METHODS

The updated version of PLSDB provides easier access to sequence files, additional visualizations, further data export options and other improvements. We want to highlight two major changes for user interaction and two changes impacting the contents of the database.

### Plasmid data collection

PLSDB is prohibitively large to manually curate and is steadily growing. Based on frequent-user feedback, two additional filtering rules were added to satisfy new community demands. The first rule is a simple threshold cutoff constraining the minimal size of a sequence to be considered a plasmid. This is necessary since the data retrieval rules set by Orlek et al. require sequences to be complete, yet smaller sequences not surpassing this threshold were observed to indicate incorrect labeling. The second rule was implemented to address incomplete assemblies and is a result of the computation of the Mash distance. The underlying designation of the Jaccard index as in Mash computation does not aim to test inclusion properties. Even in the case of perfectly covered subsequences, the set similarity threshold may not be reached leading to a retainment of both sequences. In order to address this issue, another filtering step was set in place specially focused to capture these hierarchical relationships. To this end, a blastn search querying for exact matches between plasmid pairs of the same Biosample or the same Nucleotide database description was implemented. Hereby, we considered that plasmid matches may split into two perfect matches, due to the linearization of circular plasmids in file formats. In addition to the new filtering rules, new annotations were integrated into the data collection pipeline. First, disease information from the BioSample database is now supported. Second, MOB-typer (18) has been added to annotate mobility families and mating pair formation classes.

### Annotation preprocessing

While the NCBI BioSample database offers highly relevant additional information for data analysis, it is widely accepted that the quality of the annotated meta data is lacking in several ways (25). This is because the database does not constrain meta data input upon submission. While this simplifies and encourages data upload, it complicates data analysis for users (26). Data preprocessing fixing typographical errors, annotations running under an incorrect header, incorrectly typed values, etc. is a time-consuming procedure. Yet, skipping it may negatively impact downstream analysis (25). Accordingly, a first step users often had to take when trying to make further use of the meta data of PLSDB, was to clean it up. In this update, we integrated a part of this process into our data collection pipeline, to shift some of that workload away from our users. With this goal in mind, additional processed annotations were added, while also leaving the original meta data intact. The first column we support in the new workflow indicates the host of the biosample. Here, we try to link the entry to a valid NCBI taxonomy entry. If an entry has already been resolved, we recycle the mapping. If this is not the case, we first split the text at any common separating characters, and then query the individual components in the NCBI taxonomy browser using the taxize (27) R package. If an assignment was uniquely mapped to a taxonomy, we consider it to be processed. For each biosample, we start from the host description and proceed with the isolation source column in case no result is found. The second meta data type we process indicates a potential disease of the host. As reference terminology, we use the Disease Ontology (28). Here, we start again by removing various separating characters, numerals, and stop words in the ontology terms. Afterward, we compute the case insensitive complete Levenshtein distance ratio between all terms and the query using the fuzzywuzzy python package (https://github.com/seatgeek/fuzzywuzzy) and keep the best match. Once the result surpasses a given threshold, we keep the result, for which we found a threshold of 80 to work well. In case the threshold is not met, we compute the token set ratio instead and retain the best match surpassing a threshold of 95.

### Sequence comparison

PLSDB allows a user to compare their sequences against the database choosing among a variety of search strategies. While it is useful to filter and sort detected plasmids, the displayed similarity scores may be perceived as abstract and unintuitive. In case a deeper understanding of the results had been desired, downloading of sequences and a manual investigation was previously necessary. As to improve user experience, PLSDB now allows users to visualize similarities directly and interactively in the web interface as a bipartite graph. To this end, a blastn search is run and visualization is made using Kablammo (29). Kablammo allows for filtering of blastn results to display only the most relevant similarities by adjusting blastn cutoff values. The same view can also be used to compare two selected plasmids included in the database. Further, a tblastn search is used if a user uploads protein sequences.

### Application programming interface

In the age of massively parallel sequencing automation and reproducibility is key in bioinformatics. Accordingly, PLSDB now provides an application programming interface (API). The main functionalities are focused on data retrieval and automated sequence search. A user may download information for a specific plasmid, filter the entire database for relevant subsets, or get sequence information. Users can upload their sequences through the API and remotely search for sequence similarities in PLSDB.

The API complies with OpenAPI guidelines and is further extended by an open-source python wrapper for straightforward integration into custom workflows (30). Similarly, a wrapper based on reticulate allows portability to R applications. For data analyses exceeding the throughput of the API, the open data policy of PLSDB offers the user to freely download the entire database as well as any matching sequence information.

## RESULTS

### Content summary

Since the release of PLSDB, the number of contained sequences has been continuously growing by ∼250% from the initial 13 789 to now 34 513 entries (Figure 1A). The current update consists of 350 Mb in sequence information. Focusing only on those sequences where geolocation information is available, substantial portions of the data come from China (21%), USA (21%) and the UK (8%). On the South American and African continents, Mexico and Egypt provide most of entries with 1% and less than 1%, respectively. Further, the sequences are not only sampled unevenly from a geolocational perspective, but also on a taxonomic level (Figure 1B). The phylum with most entries in PLSDB is *Proteobacteria* at 70%. Largely this is due to *Escherichia coli* species, making up ∼29% of all *Proteobacteria*, being the most predominantly represented species in the database, likely due to its model organism status. The second leading phylum, *Firmicutes*, has only a proportion of 30% compared to *Proteobacteria* with the most prevalent species being *Enterococcus faecium*. The largest relative growth compared to the first version is observed in *Actinobacteria*. Due to a low presence in the first version, relative growth is over 300-fold with a little more than one thousand entries in the current version. The least represented phyla in the current version are *Synergistetes* with one and *Chlorobi*, *Deferribacteres*, *Gemmatimonadetes*, and *Nitrospirae* with two plasmids, respectively. We observe on the gene level that the number of annotated sequences where genes involved in antimicrobial resistance are observed is growing faster than the overall number of plasmids (Figure 1C). The underlying cause for this observation may not necessarily be due to a spreading of antibiotic resistance genes. A confounding factor influencing this numerical growth might be attributed to a stronger focus of researchers on clinically relevant plasmids. In contrast to antibiotic resistances, virulence annotations decrease in relative frequency. Finally, manual analysis of the new annotation preprocessing feature shows the quality of processed annotations to be robust (Figure 1D). For the host disease meta data, available information is sparse with only 4368 entries. With our preprocessing pipeline, we were able to link 1795 entries to a valid Disease Ontology term. Considering host information, a total of 13 050 entries originally contained annotations. Taken together, we provide 12 877 processed terms, where 602 annotations derived information, despite an initially empty meta data field. Nevertheless, we note that there remain many plasmids where the automated annotation was not reliable enough to link either an ontology or taxonomy term. Here, no processed annotation is given, leaving users the choice to modify, transcribe, or drop original annotations. To gauge the overall quality of the database we assessed all sequences with various external tools used for differentiating sequences into plasmid- or chromosome-derived sequences. PlasClass (31), PlasFlow (32) and PlasForest (33) labeled 91%, 82% and 100% of sequences as plasmids respectively, indicating low contamination from e.g., chromosomal information. We do note that for PlasClass we used a threshold of 0.5 and that the tool was trained with an older version of PLSDB, likely biasing these results. To explore the completeness of the database, we compared contents to alternative databases with CD-HIT-EST-2D v4.8.1 (34). We found that 94% and 92% of sequences from the database of Brooks et al. and COMPASS, respectively were replicated in PLSDB at a sequence identity of 100%.

### Case example analysis

PLSDB is frequently used in a wide range of antimicrobial resistance-focused analyses as reference material (35). When observing antimicrobial resistance in a clinical scenario metagenomic sequencing and assembly of whole samples may not be desired due to cost or time constraints. Instead, it may be more interesting to narrow down the resistance to a few potential candidate plasmids responsible for the resistance, then to either validate it experimentally with PCR or adjust treatment (36). For demonstration purposes, we investigate a resistance qualification in *Staphylococcus aureus*, which is infamous for e.g., methicillin resistance (37). For the analysis, we search in PLSDB for available data by filtering for known plasmids in *Staphylococcus aureus*. Further, we may narrow down our findings by using the geolocational information available in PLSDB. Therefore, we filter for results originating from Germany. The number of potential plasmid candidates is now reasonably small to investigate individual plasmids. Since longer plasmids have better odds for containing an interesting gene, we sort by length and check plasmids iteratively for resistances using the plasmid overview of PLSDB. Using the newly added minimum spanning tree visualization, we may navigate among similar plasmids searching for a viable candidate, once a first interesting plasmid is identified. With few candidates selected, further investigation can be done by direct comparison. Following this general analysis gist for demonstration, we quickly identified two pivotal plasmids, NZ_CP022909.1 and NZ_CP022907.1, harboring several β-lactam resistance genes.

## CONCLUSION AND FUTURE DIRECTION

With this update, we aim to prepare PLSDB from a widely accepted data resource to a recognized reference database in the field of naturally occurring plasmids. The added features address both power and casual users alike and with them, we hope to invite more researchers into the analysis of plasmids and therein included clinically relevant resistances. Further, with the new processed meta data and content improvements to PLSDB, existing users will find important quality-of-life changes. At last, changes in the data gathering pipeline will allow us to provide regular content updates to the database for an extended amount of time.

Upcoming development efforts on the web-server will be invested into speeding up existing functionalities that may be affected by the rapid data growth such, as the search by sequence. Considering the database, future work is centered around the improvement of annotations and quality assurance of regularly scheduled data releases. Qualitatively, the goal is to further improve annotation processing by extending the natural language processing techniques with manual curation of the most frequent correction issues. Quantitatively, we aim to add more specific information if desired by the community while still balancing the user experience for new researchers entering the field. To facilitate and further automate the rolling out of future releases, we advocate for automated outlier detection in our data generation pipeline. We observed that more advanced methods were unable to provide a concise decision on plasmid classification. Yet, rule-based methods would already be able to signal suspicious annotations and potentially chromosome-contaminated sequences.

We will continuously adapt our web-server to the needs of the research community and provide accurate plasmid information in future data updates. We therefore encourage users to remain vocal on data quality and feature requests.

## DATA AVAILABILITY

The PLSDB web-server is freely accessible at: https://www.ccb.uni-saarland.de/plsdb. The entire data collection of PLSDB can be found on the website. The dedicated python package for API access is available on GitHub https://github.com/CCB-SB/plsdbapi. Finally, the data collection pipeline can be found on GitHub https://github.com/VGalata/plsdb where we are also welcoming any user feedback.
